# Injury mechanisms and occurrence locations among hospital-recorded cases in Lanshan District, Linyi, China, 2021–2025

**DOI:** 10.3389/fpubh.2026.1907077

**Published:** 2026-09-15

**Authors:** Yong Ding, Yu Liu, Zhaoxia Wang, Qi Cao, Weiqing Song

**Affiliations:** 1 Key Laboratory of Birth Defect Prevention and Control, Women and Children’s Hospital of Linyi, Linyi, Shandong, China; 2 Linyi Lanshan District Center for Disease Control and Prevention, Linyi, Shandong, China; 3 Department of Public Health, Linyi Traditional Chinese Medicine Hospital, Linyi, Shandong, China

**Keywords:** cluster influence, hospital-recorded injury visits, injury mechanism, injury surveillance, multinomial logistic regression, occurrence location

## Abstract

**Objective:**

To describe the distribution of injury mechanisms and occurrence locations among hospital-recorded injury cases and assess their associations with recorded demographic and contextual characteristics.

**Methods:**

In this retrospective study, we analyzed routinely collected injury-surveillance records from 22 reporting units in Lanshan District, Linyi, China. Records from 2021 through 25 November 2025 were eligible. After excluding four records with sex coded as unknown/other, one structurally malformed record, and one exact duplicate, 68,311 records remained. Mechanism and occurrence location were classified into seven and eight mutually exclusive categories, respectively. Multinomial logistic regression estimated relative risk ratios (RRRs) for category comparisons, adjusted for sex, age, occupation, calendar year, season, injury intent, and documented alcohol and psychotropic-substance status. Leave-one-reporting-unit-out analyses assessed the stability of the main comparisons. Modified Poisson models with reporting-unit-clustered standard errors and Cramer’s *V* were used in sensitivity and descriptive analyses.

**Results:**

Males contributed 40,433 records (59.19%). Blunt injuries were the most common mechanism (52,468; 76.81%); residences (29,529; 43.23%), roads/streets (22,346; 32.71%), and industrial/construction locations (10,829; 15.85%) were the most common locations. The principal demographic comparisons were broadly consistent across reporting-unit deletion analyses. Males had higher sharp-versus-blunt (RRR 2.02, 95% CI 1.45–2.82) and industrial/construction-versus-residence RRRs (RRR 2.68, 95% CI 2.27–3.18), and a lower road/street-versus-residence RRR (RRR 0.87, 95% CI 0.83–0.92). Compared with adults aged 35–64 years, those aged 15–34 years had higher sharp-versus-blunt (RRR 1.61, 95% CI 1.02–2.53) and road/street-versus-residence RRRs (RRR 1.41, 95% CI 1.16–1.70). Alcohol and psychotropic-substance estimates were imprecise and sensitive to reporting-unit removal.

**Conclusion:**

Sex and age were associated with the composition of mechanisms and locations among hospital-recorded injury cases. The findings describe the surveillance case series and do not estimate population incidence or causal effects. Behavioral indicators should be interpreted as exploratory because positive records were sparse and disproportionately concentrated in the largest reporting unit.

## Introduction

Injuries remain a major cause of premature mortality, disability, emergency care use, and economic loss worldwide (1–4). The distribution of injury mechanisms and settings varies by age, sex, occupation, and local social and physical environments. In China, demographic change, urbanization, motorization, and differences in occupational and residential exposure have contributed to heterogeneous injury patterns across populations and settings (3, 5). Recent multicenter and hospital-based studies have similarly shown that injury profiles differ substantially by life stage and clinical setting (6–9). Contemporary Chinese studies of courier injuries, road-traffic injuries, and in-home childhood injuries further illustrate how injury patterns vary across occupational, mobility, and domestic settings (10–12).

Hospital-based injury-surveillance systems provide timely information on patients who reach participating health facilities and can identify priorities for clinical preparedness and local prevention (13–15). However, these systems do not usually capture untreated injuries, prehospital deaths, or patients treated outside the network. Without a defined catchment population or a stable population denominator, the resulting counts cannot be interpreted as regional incidence. Changes in reporting-unit participation or data capture can also alter annual recorded volumes independently of changes in the underlying population burden.

Previous analyses of surveillance data have often focused on frequency distributions or separate binary models for selected outcomes. Injury mechanism and occurrence location are multicategory variables, so one-vs-rest models do not show how the categories compare within the same case series. Common binary outcomes also make prevalence ratios more readily interpretable than odds ratios, while records from the same institution may be correlated. Behavioral fields such as alcohol and psychotropic-substance status require attention to missingness, sparse cells, and differences in recording practice (16–20).

Using injury records from 22 reporting units in Lanshan District, Linyi, this study aimed to (1) describe the demographic, temporal, mechanism, and occurrence-location composition of recorded injury visits; (2) examine associations of recorded characteristics with seven-category injury mechanism and eight-category occurrence location using multinomial regression; and (3) assess the influence of reporting-unit composition, effect-measure choice, and behavioral-data definitions on the main comparisons. The analysis concerns differences in case composition among recorded injured patients.

## Methods

### Study design and data source

This retrospective observational study used routinely collected records from a hospital-based injury-surveillance system in Lanshan District, Linyi, Shandong Province, China. The system included 22 participating reporting units (healthcare facilities in the district sentinel injury surveillance network) during the study period. Available fields covered the reporting unit, demographic characteristics, occupation, injury timing, intent, mechanism, occurrence location, visit status, clinical outcome, and documented alcohol and psychotropic-substance information. The analysis used the complete extract supplied for the study period. Records outside participating units, external population registries, and person-level linkage to other databases were unavailable.

The earliest eligible year was 2021. Injury or visit dates extended through 25 November 2025, and data entry extended through 26 November 2025. For analysis, 2025 was treated as a partial year. When the injury-occurrence date was unavailable, the first valid date was selected in the following order for temporal classification and age derivation: visit date, entry date, and form-completion date.

The dataset did not provide a defined population catchment, population denominator, complete information on hospital level or referral pathways, or ascertainment of injuries not recorded by participating units. The analyses consequently describe recorded visits, not population incidence.

### Record selection and data cleaning

The source file used for the present analysis originally contained 68,317 records. After excluding four records with sex coded as unknown/other, one structurally malformed record, and one exact duplicate, the final analysis set contained 68,311 records (Figure 1). The difference from the 68,313 records stated in the ethics application reflects the difference between the initial record count documented during protocol approval and the final extracted analytic file after data preparation and quality screening.

**Figure 1:**
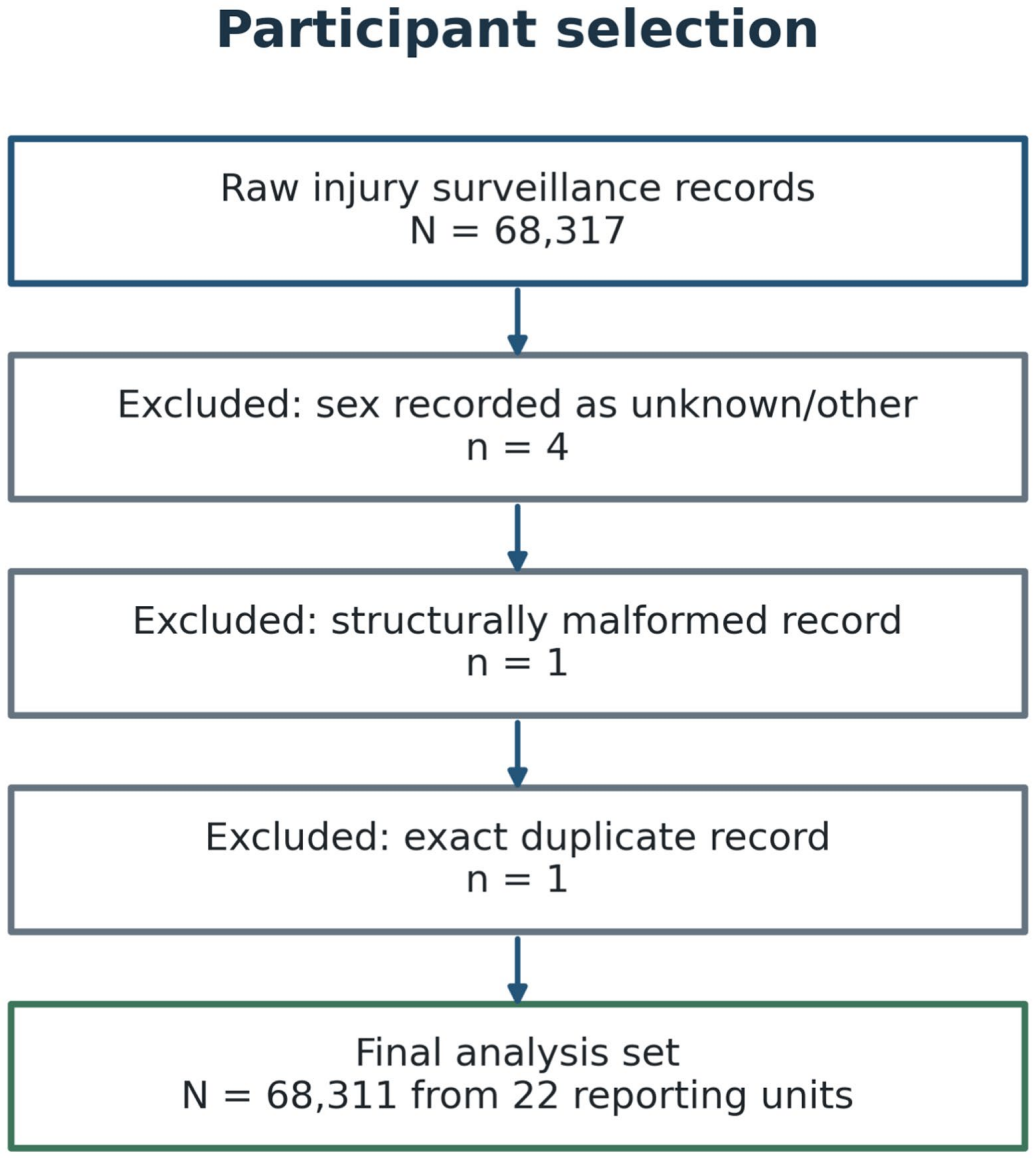
Selection of records for the final analysis.

### Variable definitions

#### Injury mechanism

The primary recorded mechanism was grouped into seven mutually exclusive categories: blunt injury, sharp injury, other mechanical injury, chemical/substance exposure, overexertion, thermal mechanism, and other mechanism. The “other” group included all remaining specified, unspecified, respiratory-threat, medical-care-complication, natural-force, and uncommon free-text entries. Blunt injury was the reference category in multinomial models.

#### Occurrence location

Occurrence location was grouped into eight categories: residence, road/street, industrial/construction location, school/educational setting, commercial setting, agricultural-production setting, recreation/sports setting, and other location. Recreation/sports combined entertainment or cultural venues, public buildings, and sports or exercise settings. Residence was the reference category in multinomial models.

#### Demographic and contextual variables

Sex was analyzed as administratively recorded male or female, with female as the reference. Age was grouped as 0–14, 15–34, 35–64, and ≥65 years, with 35–64 years as the reference. The recorded age group was used when available. For 405 records with a missing recorded age group, age was derived from date of birth and the event date; no final age-group values were missing. Agreement between recorded and date-derived groups was 99.99% among records with both sources.

Occupation was grouped using text rules into agriculture-related, commercial/service, student/child, unemployed/not working, professional/technical, and other; “other” was the model reference. These broad categories represent recorded occupational labels, not measured occupational exposure.

Calendar year was modeled categorically in multinomial analyses. Season was classified as spring (March–May), summer (June–August), autumn (September–November), or winter (December–February). Injury intent was grouped as unintentional, assault, self-harm, or undetermined/other.

Documented alcohol status was classified as no documented use by the injured person, documented use by the injured person, or missing/other-person information. Documented psychotropic-substance status was classified in the same way. The database did not identify whether these entries came from self-report, clinical observation, toxicological testing, police records, or a uniform institution-level protocol. They were treated as exploratory recorded indicators, not confirmed biological exposures.

### Statistical analysis

Categorical variables were summarized as frequencies and percentages. Pearson chi-square tests evaluated unadjusted associations with mechanism and location; bias-corrected Cramer’s V was reported to emphasize association magnitude in this large dataset. Annual recorded counts, active reporting-unit counts, the largest-unit share, and the Herfindahl–Hirschman concentration index (HHI) were described. Annual changes were interpreted only as changes in recorded case volume and composition.

Two multinomial logistic models were fitted: a seven-category mechanism model with blunt injury as the reference and an eight-category location model with residence as the reference. Both models included sex, age group, occupation, documented alcohol status, documented psychotropic-substance status, calendar year, season, and injury intent. Models were estimated with the limited-memory Broyden–Fletcher–Goldfarb–Shanno (L-BFGS) algorithm. Relative risk ratios (RRRs) were calculated by exponentiating coefficients. These multinomial models constituted the primary inferential analyses; modified Poisson and GEE models were performed only as sensitivity analyses and were not used for the main interpretation.

The main comparisons were sharp versus blunt injury, road/street versus residential location, and industrial/construction versus residential location. Records were clustered within 22 reporting units, and the full multinomial covariance matrix was rank-limited by the number of clusters. We consequently evaluated stability and uncertainty for these comparisons with leave-one-reporting-unit-out jackknife estimates. Because 22 reporting units were available, sequential removal of each unit generated 22 refits for each multinomial model, resulting in 44 refits across the two models. We also fitted reporting-unit fixed-effect multinomial models as sensitivity analyses.

Modified Poisson regression with a log link and reporting-unit-clustered standard errors was used for binary indicators of sharp injury, road/street location, and industrial/construction location (21, 22). These parsimonious models included sex, age, occupation, documented behavioral indicators, linear calendar-year trend, season, and intent. Generalized estimating-equation models with the same covariate specification and an exchangeable working correlation were fitted as a second sensitivity analysis.

Behavioral-data sensitivity analyses compared missing-indicator models, records with definitive injured-person status, and definitions based on any involved person’s documented use. Additional analyses excluded the largest reporting unit, restricted temporal analyses to 17 continuously reporting units, and restricted industrial-location models to patients aged ≥15 years who were not coded as students/children and to a working-age active-role proxy sample. Clinical outcome and non-first-visit status were examined only as post-event case-mix indicators, not as validated measures of injury severity or referral.

Diagnostics covered effective analysis N, category counts, convergence, finite coefficients and standard errors, variance inflation factors (VIFs), covariance rank, McFadden pseudo-*R*^2^, Akaike information criterion (AIC), Bayesian information criterion (BIC), sparse cells, and reporting-unit influence. Reporting-unit deletion was prioritized because dependence and recording heterogeneity operated at that level. Multiclass log loss and Brier scores were calculated descriptively. Calibration and discrimination were not interpreted as inferential diagnostics because the models estimated associations rather than a prediction tool and no external validation sample was available. Two-sided tests used α = 0.05. Interpretation emphasized effect magnitude, uncertainty, and cross-unit consistency. Analyses were conducted in Python using pandas, SciPy, and statsmodels.

### Reporting guideline

Reporting followed the STROBE statement and the RECORD extension for studies using routinely collected health data (16, 17).

### Ethics

The study protocol, including the waiver of informed consent, was approved by the Medical Ethics Committee of Linyi Traditional Chinese Medicine Hospital on 22 July 2025 (approval no. 20250082). The waiver was granted because this retrospective study analyzed de-identified routinely collected surveillance records, involved no intervention, and posed no more than minimal risk to participants. The approved protocol covered the planned surveillance period through December 2025; the available analytic dataset contained records entered through 26 November 2025, so the analysis was restricted accordingly.

## Results

### Sample characteristics

The final analysis included 68,311 recorded injury visits from 22 reporting units (Figure 1). Males accounted for 40,433 records (59.19%). Adults aged 35–64 years represented 52.91% of records, followed by adults aged ≥65 years (21.00%), those aged 15–34 years (17.87%), and children aged 0–14 years (8.21%). Agriculture-related occupations were recorded in 46.23% of visits. Unintentional injuries accounted for 95.67% (Table 1).

**Table 1:** Characteristics of hospital-recorded injury visits (*N* = 68,311).

**Variable**	**Category**	*n*	**%**
Sex	Female	27,878	40.81
	Male	40,433	59.19
Age group	0–14 years	5,611	8.21
	15–34 years	12,206	17.87
	35–64 years	36,146	52.91
	≥65 years	14,348	21.00
Occupation	Agriculture-related	31,580	46.23
	Commercial/service	4,355	6.38
	Student/child	6,853	10.03
	Unemployed/not working	4,696	6.87
	Professional/technical	5,073	7.43
	Other	15,754	23.06
Calendar year	2021	9,467	13.86
	2022	9,261	13.56
	2023	11,432	16.74
	2024	19,122	27.99
	2025 (partial year)	19,029	27.86
Season	Spring	18,516	27.11
	Summer	19,654	28.77
	Autumn	16,529	24.20
	Winter	13,612	19.93
Injury intent	Unintentional	65,351	95.67
	Assault	1,701	2.49
	Self-harm	690	1.01
	Undetermined/other	569	0.83
Injured-person alcohol status	No documented use	66,705	97.65
	Documented use	330	0.48
	Missing/other person	1,276	1.87
Injured-person psychotropic status	No documented use	66,641	97.56
	Documented use	365	0.53
	Missing/other person	1,305	1.91

Documented injured-person alcohol use was recorded in 330 cases (0.48%), and documented injured-person psychotropic-substance use in 365 (0.53%). Missing/other-person information represented 1.87 and 1.91%, respectively. These positive records were not evenly distributed: the largest reporting unit contributed approximately 69.7% of alcohol-positive and 84.7% of psychotropic-positive records.

### Injury-mechanism and occurrence-location composition

Blunt injuries were the most frequently recorded mechanism (52,468; 76.81%), followed by sharp injuries (5,692; 8.33%) and other mechanical injuries (5,309; 7.77%) (Table 2). Residences accounted for 29,529 records (43.23%), roads/streets for 22,346 (32.71%), and industrial/construction locations for 10,829 (15.85%) (Table 3).

**Table 2:** Injury-mechanism composition (*N* = 68,311).

**Mechanism**	***n***	**%**
Blunt injury	52,468	76.81
Sharp injury	5,692	8.33
Other mechanical injury	5,309	7.77
Chemical/substance exposure	1,568	2.30
Overexertion	1,519	2.22
Thermal mechanism	941	1.38
Other mechanism	814	1.19

**Table 3:** Occurrence-location composition (*N* = 68,311).

Occurrence location	*n*	%
Residence	29,529	43.23
Road/street	22,346	32.71
Industrial/construction location	10,829	15.85
Recreation/sports setting	1,372	2.01
Other location	1,229	1.80
School/educational setting	1,144	1.67
Commercial setting	1,010	1.48
Agricultural-production setting	852	1.25

Figure 2 shows age- and sex-stratified percentages within the recorded case series. Sharp and industrial/construction categories comprised larger shares among younger and middle-aged males, whereas residential locations comprised a larger share among older adults.

**Figure 2:**
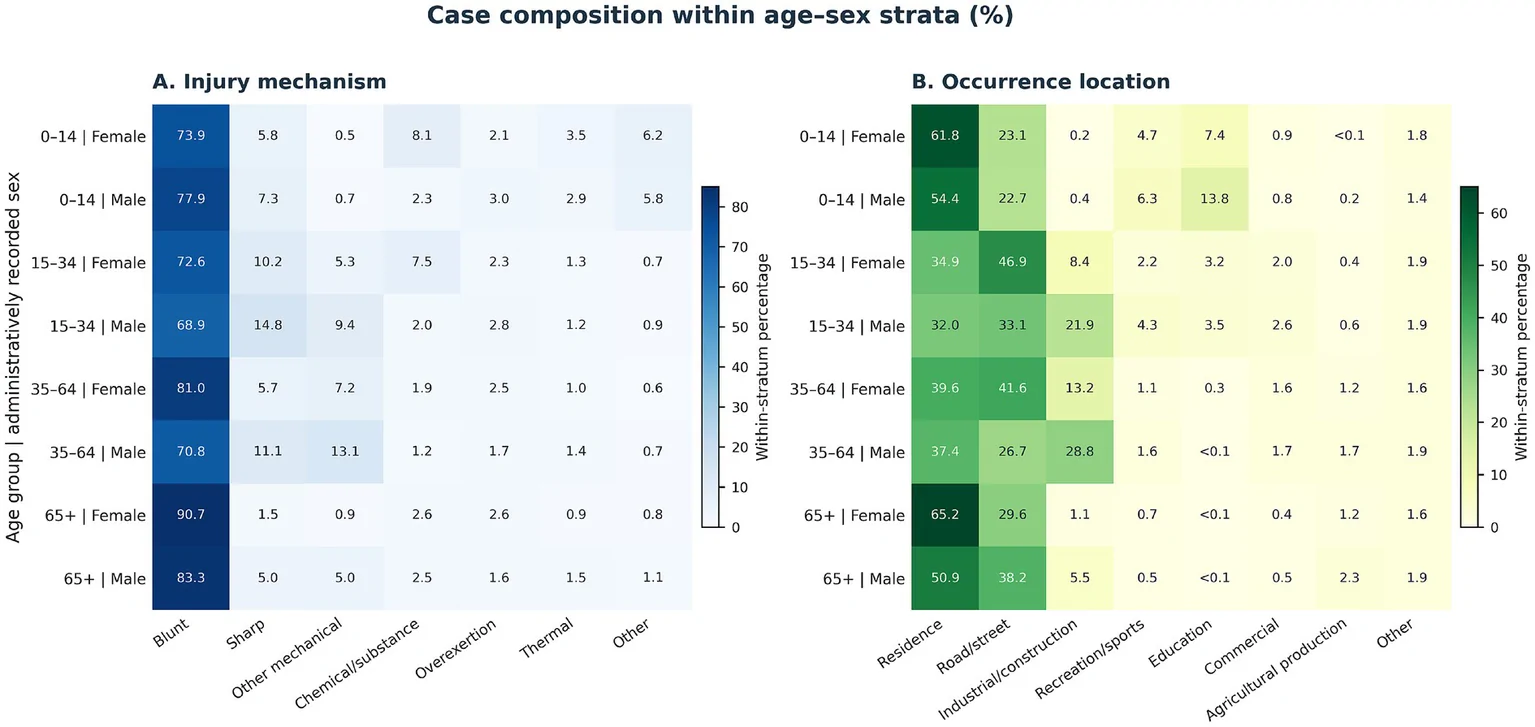
**(A)** Injury-mechanism and **(B)** occurrence-location composition within age–sex strata. Each row sums to 100% within the corresponding outcome family.

### Surveillance profile and unadjusted associations

Annual recorded visits were 9,467 in 2021, 9,261 in 2022, 11,432 in 2023, 19,122 in 2024, and 19,029 during the partial 2025 observation period. Active reporting units varied from 17 to 21. The largest unit’s annual share declined from 69.45% in 2021 to 42.70% in 2025, and the HHI declined from 0.496 to 0.224 (Figure 3). Seventeen units reported in every calendar year and contributed 67,935 records (99.45%). Annual prevalence-ratio estimates from this continuous network were almost unchanged from the full-network estimates. Year-specific n (%) for all seven mechanisms and eight locations are reported in Supplementary Table S12. The annual figures describe recorded volume and case composition within a changing reporting network.

**Figure 3:**
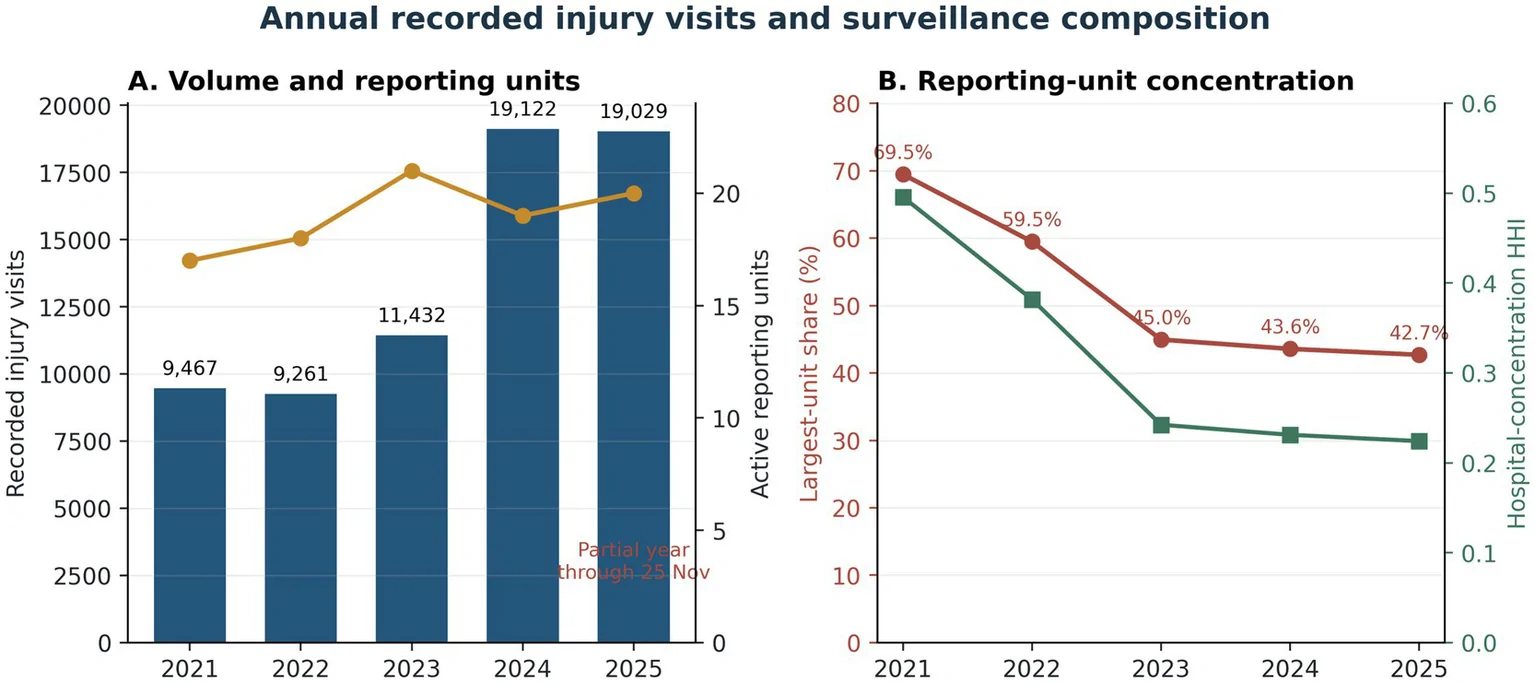
Annual recorded injury visits, active reporting units **(A)**, and reporting-unit concentration **(B)**. The 2025 data extend through 25 November.

Chi-square tests were statistically small in relation to the sample size, while effect sizes varied. Injury intent had the largest association with mechanism composition (bias-corrected Cramer’s *V* = 0.392). Associations of mechanism with year (*V* = 0.062) and season (*V* = 0.038) were comparatively weak. For occurrence location, intent (*V* = 0.230) and age group (*V* = 0.230) had the largest Cramer’s *V* values; associations with year (*V* = 0.045) and season (*V* = 0.047) were weak. Complete effect-size results are provided in Supplementary Table S3.

### Multinomial associations

Both multinomial models converged with finite coefficients and standard errors. In the sharp-versus-blunt comparison, the RRR was higher for males (RRR 2.02, reporting-unit jackknife 95% CI 1.45–2.82). Compared with age 35–64 years, the RRR was higher for age 15–34 years (RRR 1.61, 95% CI 1.02–2.53) and lower for age ≥65 years (RRR 0.32, 95% CI 0.28–0.36).

In the road/street-versus-residence comparison, the RRR was lower for males (RRR 0.87, 95% CI 0.83–0.92). Compared with age 35–64 years, the RRR was higher for age 15–34 years (RRR 1.41, 95% CI 1.16–1.70) and lower for age ≥65 years (RRR 0.66, 95% CI 0.55–0.79).

In the industrial/construction-versus-residence comparison, the RRR was higher for males (RRR 2.68, 95% CI 2.27–3.18). Compared with age 35–64 years, the RRRs were lower for ages 0–14 years (RRR 0.045, 95% CI 0.018–0.112) and ≥65 years (RRR 0.109, 95% CI 0.072–0.164). The child estimate reflects the age structure of access to industrial workplaces (Table 4 and Figure 4).

**Table 4:** Multinomial associations stable across reporting-unit jackknife analyses.

Outcome comparison	Characteristic	Reference	RRR	Reporting-unit jackknife 95% CI
Sharp vs. blunt	Male	Female	2.022	1.451–2.818
Sharp vs. blunt	Age 15–34 years	Age 35–64 years	1.606	1.021–2.527
Sharp vs. blunt	Age ≥65 years	Age 35–64 years	0.317	0.280–0.359
Road/street vs. residence	Male	Female	0.873	0.832–0.917
Road/street vs. residence	Age 15–34 years	Age 35–64 years	1.409	1.164–1.705
Road/street vs. residence	Age ≥65 years	Age 35–64 years	0.659	0.550–0.788
Industrial/construction location vs. residence	Male	Female	2.683	2.267–3.176
Industrial/construction location vs. residence	Age 0–14 years	Age 35–64 years	0.045	0.018–0.112
Industrial/construction location vs. residence	Age ≥65 years	Age 35–64 years	0.109	0.072–0.164

**Figure 4:**
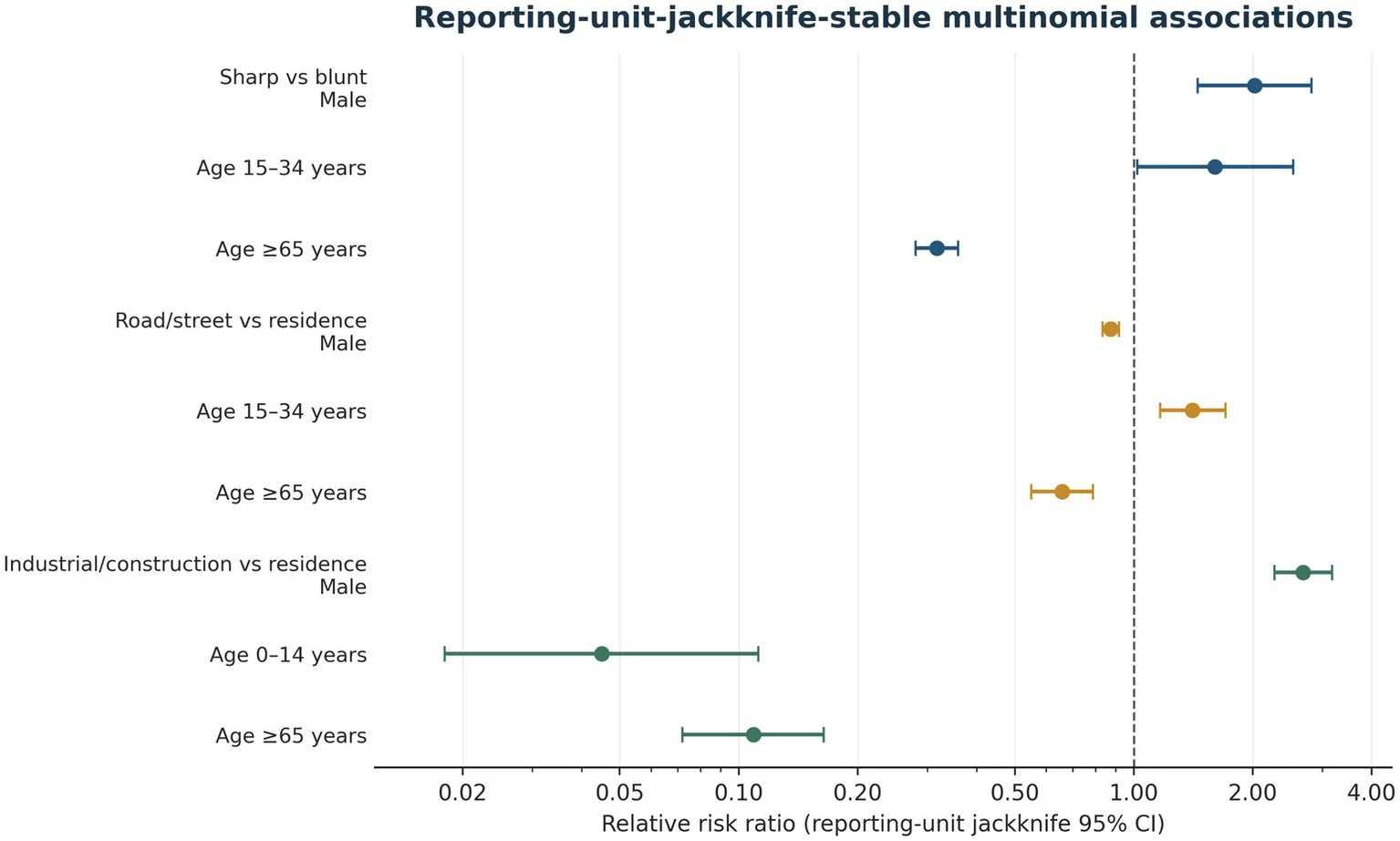
Multinomial associations stable across leave-one-reporting-unit-out analyses.

RRR, relative risk ratio. “Industrial/construction location” denotes the industrial/construction occurrence-location category. Models adjusted for sex, age group, occupation, documented alcohol status, documented psychotropic-substance status, calendar year, season, and injury intent. The 95% CIs were obtained from leave-one-reporting-unit-out jackknife estimates.

### Sensitivity and model diagnostics

The parsimonious modified Poisson and matched exchangeable-correlation GEE models all converged. Modified Poisson models had full-rank clustered covariance matrices and no predicted values above one. The principal demographic estimates were similar across methods. For males, the modified Poisson and GEE PRs were 1.78 and 1.78 for sharp injury, 0.77 and 0.81 for road/street location, and 2.25 and 2.07 for industrial/construction location. Corresponding estimates and 95% CIs for the prespecified comparisons are reported in Supplementary Tables S4, S10.

Behavioral-exposure estimates varied across reporting-unit sensitivity analyses. For example, the full multinomial RRR for documented injured-person alcohol use in the sharp-versus-blunt comparison was 2.50, with a jackknife 95% CI of 0.18–34.33; the estimate changed direction after removal of the largest unit. In the road/street-versus-residence comparison, the alcohol jackknife 95% CI was 0.13–7.26. Documented psychotropic-substance estimates were similarly imprecise. Category-specific positive counts and the number of contributing reporting units are reported in Supplementary Table S11. These findings are presented as exploratory results (Supplementary Figure S1).

The leave-one-reporting-unit-out procedure generated 44 refits; together with the two full-sample models, all 46 multinomial model fits converged. Among 33 main coefficients, 11 changed direction at least once. The industrial/construction coefficient for commercial/service occupation was particularly unit-dependent: the full-sample RRR was 2.90, the jackknife 95% CI was 0.032–265.82, and removal of the largest unit yielded an RRR below one. This comparison was not treated as a consistent finding.

Restricting industrial-location analyses to patients aged ≥15 years who were not classified as students/children (*N* = 60,702) and to a working-age active-role proxy sample (*N* = 43,542) produced converged, full-rank modified Poisson models. These results are consistent with the student/child comparison reflecting structural non-exposure, not an independently protective characteristic.

### Model diagnostics

Both multinomial models used all 68,311 records, with no additional exclusions for missing covariates. The seven-category mechanism model converged after 200 L-BFGS iterations and had McFadden pseudo-*R*^2^ = 0.1315, AIC = 106,055.3, and BIC = 107,370.3; its smallest category contained 814 records. The eight-category location model converged after 316 iterations and had pseudo-*R*^2^ = 0.1191, AIC = 163,561.8, and BIC = 165,095.9; its smallest category contained 852 records. Both models had finite coefficients and standard errors. VIFs for age and occupation indicators were below 3; the largest values, approximately 5.5, involved overlapping missing-status indicators for alcohol and psychotropic-substance fields. Diagnostics relevant to estimation, sparsity, and reporting-unit influence are provided in Supplementary Tables S5–S7, S11.

## Discussion

### Principal findings

This analysis of 68,311 hospital-recorded injury visits found that blunt mechanisms and residential and road/street settings comprised most recorded cases. Sex and age were associated with the relative composition of selected mechanisms and locations after adjustment for available covariates and assessment of reporting-unit influence. Within the recorded case series, males had higher sharp-versus-blunt and industrial/construction-versus-residence RRRs and a lower road/street-versus-residence RRR. Younger adults had higher sharp-versus-blunt and road/street-versus-residence RRRs, whereas older adults had lower RRRs in the main comparisons. These associations describe case composition and do not establish population injury risk or causal effects.

### Interpretation of demographic patterning

The higher sharp-versus-blunt RRRs among males and younger adults are consistent with prior Chinese evidence that injury patterns vary by life stage, work-related tasks, travel context, and domestic environment (6–12). These potential explanations were not measured directly in the surveillance dataset. The higher industrial/construction-versus-residence RRR among males likewise describes the composition of recorded cases, not an occupational incidence rate, because no workforce denominator or task-specific exposure information was available.

The lower industrial/construction-versus-residence RRRs among children and older adults are consistent with their lower routine access to industrial workplaces. The adult and active-role restriction analyses reduced this overlap but did not create a true occupational risk set, so the child and student comparisons should not be interpreted as protective effects.

The road/street-versus-residence RRR below one for males indicates that road/street locations were less common relative to residences within the male case composition than within the corresponding female case composition. It is not an estimate of road-injury risk in the male population.

### Surveillance coverage and temporal interpretation

Recorded visits increased after 2022, while active-unit counts and the distribution of records across units changed. The largest reporting unit accounted for nearly 70% of records in 2021 and approximately 43% in 2025. Restriction to continuously reporting units yielded similar annual composition estimates because these units captured 99.45% of records, but this does not establish stable population coverage. The 2025 data were also incomplete. Annual findings are best used for surveillance planning and data-quality assessment, not as regional incidence trends.

Hospital data can characterize the patients and events reaching participating services, identify data-quality problems, and support local preparedness (13–15). Population incidence would require defined residency, case ascertainment across providers and prehospital deaths, and a population or service denominator.

### Behavioral indicators and missing information

Documented injured-person alcohol and psychotropic-substance use were rare and concentrated in the largest reporting unit. Estimates changed markedly when individual units were removed, and jackknife intervals were wide. The dataset could not determine whether the source was self-report, clinical observation, toxicological testing, police information, or another process. These variables should consequently be read as indicators of recorded documentation, not as measured substance exposure.

Classifying missing/other information separately preserved records but did not remove selection bias. Complete-record and alternative-definition analyses addressed different assumptions and produced variable estimates. If missingness depended on injury severity, liability context, consciousness, testing availability, or institutional practice, neither a missing indicator nor complete-case analysis would recover an unbiased causal effect without additional information (18–20, 23). Future surveillance could benefit from standardized field definitions, documented information sources, and linkage with validated clinical or laboratory data.

### Implications

The demographic patterns that were broadly consistent across reporting-unit analyses can inform descriptive surveillance summaries and hypothesis generation. Reporting systems may benefit from routine monitoring of mechanism and location composition by sex and age, accompanied by institution-level completeness indicators. Prevention programs aimed at road, residential, or workplace settings require designs with population or exposure denominators.

The Haddon Matrix remains a useful organizing framework for future work on host, agent, and environmental factors, although the present dataset covered only a subset of its domains (24, 25).

### Strengths and limitations

The study benefits from a large routinely collected case series, an auditable cleaning procedure, complete multicategory outcome distributions, multinomial primary models, prevalence-ratio sensitivity analyses, and several assessments of reporting-unit dependence and influence. Its interpretation is limited by the surveillance frame: the sample excludes injuries not recorded by participating units, and no stable catchment population or service-volume denominator was available. Reporting-unit participation and concentration changed over time, referral pathways and duplicate encounters could not be fully identified, and the findings come from a single district. Socioeconomic status, education, urbanization, comorbidities, task-specific occupational exposure, verified injury severity, and time-of-day exposure were unavailable. Mechanism, location, occupation, and behavioral indicators may be misclassified; alcohol and psychotropic-substance records were sparse and institutionally concentrated. Finally, only 22 reporting units were available for cluster-level inference, and broad occupation categories did not define true worker populations.

## Conclusion

Among hospital-recorded injury visits in Lanshan District, Linyi, the composition of mechanisms and locations differed by sex and age. Within the recorded case series, males had higher sharp-versus-blunt and industrial/construction-versus-residence RRRs, while younger adults had higher sharp-versus-blunt and road/street-versus-residence RRRs. These findings do not estimate population incidence or causal effects. Behavioral indicators were sparse and reporting-unit dependent; population-risk and intervention studies will require defined denominators, broader case ascertainment, and standardized exposure measurement.

## Data Availability

The data analyzed in this study is subject to the following licenses/restrictions: The data are not publicly available due to institutional, privacy, and data-governance restrictions. Requests to access these datasets should be directed to WS, swqlyszyy@163.com.
