# Supplementary material for: Injury mechanisms and occurrence locations among hospital-recorded cases in Lanshan District, Linyi, China, 2021–2025
Source: Front Public Health. 2026 Sep 15;14:1907077. doi: 10.3389/fpubh.2026.1907077 (PMC13619393; doi:10.3389/fpubh.2026.1907077)
Supplement: Supplementary file 1 [file Supplementary_file_1.DOCX]

Supplementary Material

# Injury Mechanisms and Occurrence Locations among Hospital-Recorded Cases in Lanshan District, China, 2021–2025

# Supplementary Table S1. Analysis-variable codebook

| **Analysis variable** | **Source field** | **Coding** | **Reference/role** |
| --- | --- | --- | --- |
| Sex | Sex | Female; male | Female reference; unknown/other excluded |
| Age group | Recorded age group; date of birth; event date | 0–14; 15–34; 35–64; ≥65 years | 35–64 reference; 405 missing recorded groups derived from dates |
| Occupation | Occupation/work type | Agriculture-related; commercial/service; student/child; unemployed/not working; professional/technical; other | Other reference; keyword-based broad groups |
| Calendar year | Injury/visit/entry/form date | 2021–2025 | Categorical in multinomial models; linear in parsimonious Poisson sensitivity models |
| Season | Event month | Spring; summer; autumn; winter | Spring reference |
| Injury intent | Injury intent | Unintentional; assault; self-harm; undetermined/other | Unintentional reference |
| Injured-person alcohol status | Alcohol-use text | No documented use; documented injured-person use; missing/other-person information | No documented use reference; exploratory |
| Injured-person psychotropic status | Psychotropic-substance text | No documented use; documented injured-person use; missing/other-person information | No documented use reference; exploratory |
| Mechanism | Primary injury mechanism | Blunt; sharp; other mechanical; chemical/substance; overexertion; thermal; other | Blunt reference |
| Occurrence location | Primary location | Residence; road/street; industrial/construction; education; commercial; agricultural production; recreation/sports; other | Residence reference |
| Reporting unit | Reporting unit | H01–H22 anonymous identifiers | Cluster and influence unit |

Occupation keywords were applied in the following order: agriculture-related (“agriculture,” farming, livestock, fishing, cultivation, breeding); student/child; commercial/service (service, business, sales, catering, security, driver); professional/technical (teacher, physician, nurse, engineering, technology, research, accounting, public service); unemployed/not working (unemployed, retired, job-seeking); and other.

# Supplementary Table S2. Complete analysis-category distributions

| **Variable** | **Category** | **n** | **%** |
| --- | --- | --- | --- |
| Age | 0–14 | 5,611 | 8.21 |
|  | 15–34 | 12,206 | 17.87 |
|  | 35–64 | 36,146 | 52.91 |
|  | ≥65 | 14,348 | 21.00 |
| Occupation | Agriculture-related | 31,580 | 46.23 |
|  | Other | 15,754 | 23.06 |
|  | Student/child | 6,853 | 10.03 |
|  | Professional/technical | 5,073 | 7.43 |
|  | Unemployed/not working | 4,696 | 6.87 |
|  | Commercial/service | 4,355 | 6.38 |
| Injury intent | Unintentional | 65,351 | 95.67 |
|  | Assault | 1,701 | 2.49 |
|  | Self-harm | 690 | 1.01 |
|  | Undetermined/other | 569 | 0.83 |
| Mechanism | Blunt | 52,468 | 76.81 |
|  | Sharp | 5,692 | 8.33 |
|  | Other mechanical | 5,309 | 7.77 |
|  | Chemical/substance | 1,568 | 2.30 |
|  | Overexertion | 1,519 | 2.22 |
|  | Thermal | 941 | 1.38 |
|  | Other | 814 | 1.19 |
| Location | Residence | 29,529 | 43.23 |
|  | Road/street | 22,346 | 32.71 |
|  | Industrial/construction | 10,829 | 15.85 |
|  | Recreation/sports | 1,372 | 2.01 |
|  | Other | 1,229 | 1.80 |
|  | Education | 1,144 | 1.67 |
|  | Commercial | 1,010 | 1.48 |
|  | Agricultural production | 852 | 1.25 |

# Supplementary Table S3. Bias-corrected Cramer’s V for unadjusted associations

| **Outcome** | **Predictor** | **Cramer’s V** | **Sparse-cell note** |
| --- | --- | --- | --- |
| Mechanism | Injury intent | 0.392 | No expected cells <5 |
| Mechanism | Psychotropic status | 0.213 | 1 expected cell <5 |
| Mechanism | Sex | 0.153 | No expected cells <5 |
| Mechanism | Age group | 0.141 | No expected cells <5 |
| Mechanism | Occupation | 0.123 | No expected cells <5 |
| Mechanism | Alcohol status | 0.108 | 2 expected cells <5 |
| Mechanism | Calendar year | 0.062 | No expected cells <5 |
| Mechanism | Season | 0.038 | No expected cells <5 |
| Location | Injury intent | 0.230 | No expected cells <5 |
| Location | Age group | 0.230 | No expected cells <5 |
| Location | Sex | 0.196 | No expected cells <5 |
| Location | Occupation | 0.184 | No expected cells <5 |
| Location | Alcohol status | 0.053 | 2 expected cells <5 |
| Location | Psychotropic status | 0.048 | 1 expected cell <5 |
| Location | Season | 0.047 | No expected cells <5 |
| Location | Calendar year | 0.045 | No expected cells <5 |

# Supplementary Table S4. Modified Poisson sensitivity analyses

| **Binary outcome** | **Characteristic** | **Adjusted PR** | **95% CI** | **Interpretation** |
| --- | --- | --- | --- | --- |
| Sharp injury | Male | 1.783 | 1.483–2.143 | Broadly consistent across analyses |
| Sharp injury | Age 15–34 | 1.520 | 1.260–1.834 | Broadly consistent across analyses |
| Sharp injury | Age ≥65 | 0.372 | 0.326–0.424 | Broadly consistent across analyses |
| Sharp injury | Documented injured-person alcohol | 1.909 | 1.160–3.141 | Reporting-unit-sensitive; exploratory |
| Sharp injury | Documented injured-person psychotropic use | 0.561 | 0.379–0.832 | Reporting-unit-sensitive; exploratory |
| Road/street | Male | 0.773 | 0.748–0.799 | Broadly consistent across analyses |
| Road/street | Age 15–34 | 1.203 | 1.141–1.268 | Broadly consistent across analyses |
| Road/street | Age ≥65 | 0.953 | 0.872–1.042 | No clear binary association |
| Road/street | Documented injured-person alcohol | 1.050 | 0.842–1.308 | No clear association |
| Road/street | Documented injured-person psychotropic use | 0.450 | 0.216–0.935 | Reporting-unit-sensitive; exploratory |
| Industrial/construction location | Male | 2.247 | 1.844–2.737 | Broadly consistent across analyses |
| Industrial/construction location | Age 0–14 | 0.073 | 0.032–0.166 | Structural non-exposure |
| Industrial/construction location | Age 15–34 | 0.856 | 0.800–0.916 | Case-composition association |
| Industrial/construction location | Age ≥65 | 0.165 | 0.145–0.189 | Broadly consistent across analyses |
| Industrial/construction location | Commercial/service occupation | 1.941 | 1.210–3.113 | Multinomial reporting-unit jackknife extremely unstable |
| Industrial/construction location | Documented injured-person alcohol | 0.364 | 0.289–0.457 | Reporting-unit-sensitive; not protective |
| Industrial/construction location | Documented injured-person psychotropic use | 0.771 | 0.531–1.119 | No clear association |

“Industrial/construction location” denotes the industrial/construction occurrence-location category. PRs were estimated from parsimonious log-link Poisson models with reporting-unit-clustered standard errors. Models included sex, age, occupation, documented alcohol and psychotropic status, linear calendar year, season, and injury intent. Matched exchangeable-correlation GEE results using the same adjustment set are reported in Supplementary Table S10.

# Supplementary Table S5. Selected multinomial reporting-unit jackknife results

| **Comparison** | **Characteristic** | **Full-sample RRR** | **Jackknife 95% CI** | **Stability assessment** |
| --- | --- | --- | --- | --- |
| Sharp vs blunt | Male | 2.022 | 1.451–2.818 | Stable |
| Sharp vs blunt | Age 15–34 | 1.606 | 1.021–2.527 | Stable; lower precision |
| Sharp vs blunt | Age ≥65 | 0.317 | 0.280–0.359 | Stable |
| Sharp vs blunt | Alcohol positive | 2.499 | 0.182–34.334 | Unstable; direction changed after removing H01 |
| Sharp vs blunt | Psychotropic positive | 1.245 | 0.452–3.433 | Unstable |
| Road/street vs residence | Male | 0.873 | 0.832–0.917 | Stable |
| Road/street vs residence | Age 15–34 | 1.409 | 1.164–1.705 | Stable |
| Road/street vs residence | Age ≥65 | 0.659 | 0.550–0.788 | Stable |
| Road/street vs residence | Alcohol positive | 0.957 | 0.126–7.265 | Unstable; direction flips |
| Road/street vs residence | Psychotropic positive | 0.292 | 0.040–2.151 | Imprecise |
| Industrial/construction location vs residence | Male | 2.683 | 2.267–3.176 | Stable |
| Industrial/construction location vs residence | Age 0–14 | 0.045 | 0.018–0.112 | Stable but structural non-exposure |
| Industrial/construction location vs residence | Age ≥65 | 0.109 | 0.072–0.164 | Stable |
| Industrial/construction location vs residence | Student/child occupation | 0.062 | 0.023–0.166 | Structural non-exposure; not protective |
| Industrial/construction location vs residence | Commercial/service occupation | 2.904 | 0.032–265.815 | Extremely reporting-unit-dependent |
| Industrial/construction location vs residence | Alcohol positive | 0.293 | 0.049–1.760 | Unstable; not protective |
| Industrial/construction location vs residence | Psychotropic positive | 0.466 | 0.203–1.068 | Imprecise |

*Supplementary Figure S1. Leave-one-reporting-unit-out influence for two stable demographic coefficients and two reporting-unit-sensitive coefficients. Circles show estimates after deleting each reporting unit, diamonds show full-sample estimates, and red crosses identify deletion of the largest unit (H01).*

# Supplementary Table S6. Multinomial model diagnostics

| **Diagnostic** | **Mechanism model** | **Location model** |
| --- | --- | --- |
| Effective analysis N (no covariate exclusions) | 68,311 | 68,311 |
| Outcome categories | 7 | 8 |
| Predictors excluding intercept | 23 | 23 |
| Total coefficients | 144 | 168 |
| L-BFGS iterations | 200 | 316 |
| Converged | Yes | Yes |
| Non-finite coefficients | 0 | 0 |
| Non-finite standard errors | 0 | 0 |
| Minimum outcome count | 814 | 852 |
| Log likelihood | -52,883.65 | -81,612.90 |
| Null log likelihood | -60,889.38 | -92,650.71 |
| McFadden pseudo-R² | 0.1315 | 0.1191 |
| AIC | 106,055.3 | 163,561.8 |
| BIC | 107,370.3 | 165,095.9 |
| Apparent log loss | 0.7742 | 1.1947 |
| Apparent multiclass Brier score | 0.3607 | 0.6310 |

Apparent classification metrics were calculated on the estimation data and are not external predictive validation.

# Supplementary Table S7. Variance inflation factors for the extended design matrix

| **Term** | **VIF** |
| --- | --- |
| Psychotropic status: missing/other | 5.517 |
| Alcohol status: missing/other | 5.504 |
| Student/child occupation | 2.959 |
| Age 0–14 | 2.732 |
| Year 2024 | 2.274 |
| Year 2025 | 2.272 |
| Year 2023 | 1.901 |
| Year 2022 | 1.731 |
| Agriculture-related occupation | 1.632 |
| Summer | 1.489 |
| Autumn | 1.460 |
| Winter | 1.403 |
| All remaining indicators | <1.25 |

# Supplementary Table S8. Reporting-unit coverage sensitivity

| **Year** | **Recorded visits** | **Active units** | **Largest-unit share (%)** | **HHI** |
| --- | --- | --- | --- | --- |
| 2021 | 9,467 | 17 | 69.45 | 0.496 |
| 2022 | 9,261 | 18 | 59.48 | 0.382 |
| 2023 | 11,432 | 21 | 44.95 | 0.242 |
| 2024 | 19,122 | 19 | 43.57 | 0.231 |
| 2025* | 19,029 | 20 | 42.70 | 0.224 |

*2025 data extend through 25 November. Seventeen continuously reporting units contributed 67,935 records (99.45%). Annual PR estimates from the full network and continuous network were 0.950 vs 0.949 for sharp injury, 1.041 vs 1.041 for road/street location, and 0.961 vs 0.961 for industrial/construction location.

# Supplementary Table S9. Structural non-exposure sensitivity for industrial/construction location

| **Analysis set** | **N** | **Events** | **Clusters** | **Selected result** | **Adjusted PR (95% CI)** |
| --- | --- | --- | --- | --- | --- |
| Age ≥15 and non-student | 60,702 | 10,793 | 22 | Male | 2.248 (1.845–2.738) |
|  |  |  |  | Age ≥65 vs 35–64 | 0.166 (0.145–0.189) |
|  |  |  |  | Commercial/service vs other | 1.940 (1.210–3.111) |
| Working-age active-role proxy | 43,542 | 10,193 | 21 | Male | 2.138 (1.735–2.636) |
|  |  |  |  | Age 15–34 vs 35–64 | 0.864 (0.806–0.927) |
|  |  |  |  | Commercial/service vs other | 1.881 (1.175–3.014) |

Both models converged with full-rank clustered covariance matrices. These restrictions do not define a true employed-population denominator and do not remove the reporting-unit dependence of selected occupation and behavioral results.

# Supplementary Table S10. Matched modified Poisson and generalized estimating-equation sensitivity analyses

| **Binary outcome** | **Characteristic** | **Clustered PR (95% CI)** | **GEE PR (95% CI)** | **GEE P** |
| --- | --- | --- | --- | --- |
| Sharp injury | Male | 1.783 (1.483–2.143) | 1.780 (1.516–2.089) | <0.001 |
| Sharp injury | Age 15–34 | 1.520 (1.260–1.834) | 1.528 (1.272–1.835) | <0.001 |
| Sharp injury | Age ≥65 | 0.372 (0.326–0.424) | 0.371 (0.331–0.415) | <0.001 |
| Sharp injury | Documented injured-person alcohol | 1.909 (1.160–3.141) | 1.914 (1.188–3.085) | 0.008 |
| Sharp injury | Documented injured-person psychotropic use | 0.561 (0.379–0.832) | 0.553 (0.383–0.799) | 0.002 |
| Road/street | Male | 0.773 (0.748–0.799) | 0.809 (0.774–0.845) | <0.001 |
| Road/street | Age 15–34 | 1.203 (1.141–1.268) | 1.176 (1.123–1.232) | <0.001 |
| Road/street | Age ≥65 | 0.953 (0.872–1.042) | 0.946 (0.864–1.036) | 0.233 |
| Road/street | Documented injured-person alcohol | 1.050 (0.842–1.308) | 1.092 (0.933–1.278) | 0.273 |
| Road/street | Documented injured-person psychotropic use | 0.450 (0.216–0.935) | 0.540 (0.336–0.869) | 0.011 |
| Industrial/construction | Male | 2.247 (1.844–2.737) | 2.072 (1.763–2.437) | <0.001 |
| Industrial/construction | Age 0–14 | 0.073 (0.032–0.166) | 0.103 (0.048–0.221) | <0.001 |
| Industrial/construction | Age 15–34 | 0.856 (0.800–0.916) | 0.862 (0.816–0.910) | <0.001 |
| Industrial/construction | Age ≥65 | 0.165 (0.145–0.189) | 0.201 (0.171–0.237) | <0.001 |
| Industrial/construction | Commercial/service occupation | 1.941 (1.210–3.113) | 1.883 (1.271–2.789) | 0.002 |
| Industrial/construction | Documented injured-person alcohol | 0.364 (0.289–0.457) | 0.379 (0.310–0.464) | <0.001 |
| Industrial/construction | Documented injured-person psychotropic use | 0.771 (0.531–1.119) | 0.730 (0.499–1.066) | 0.103 |

*Both models used the same parsimonious adjustment set: sex, age, occupation, documented alcohol and psychotropic-substance status, linear calendar year, season, and injury intent. Clustered PRs use reporting-unit-clustered standard errors; GEE models use an exchangeable working correlation across 22 reporting units. Behavioral estimates remain exploratory because positive records were sparse and concentrated by reporting unit.*

# Supplementary Table S11. Documented positive behavioral indicators within each modeled outcome category

**Panel A. Injury mechanism**

| **Category** | **Category N** | **Alcohol positive, n (%)** | **Units, n** | **Psychotropic positive, n (%)** | **Units, n** |
| --- | --- | --- | --- | --- | --- |
| Blunt injury | 52,468 | 156 (0.30) | 12 | 102 (0.19) | 12 |
| Sharp injury | 5,692 | 59 (1.04) | 5 | 15 (0.26) | 4 |
| Other mechanical injury | 5,309 | 7 (0.13) | 2 | 8 (0.15) | 2 |
| Chemical/substance exposure | 1,568 | 101 (6.44) | 2 | 223 (14.22) | 3 |
| Overexertion | 1,519 | 0 (0.00) | 0 | 3 (0.20) | 3 |
| Thermal mechanism | 941 | 4 (0.43) | 1 | 2 (0.21) | 1 |
| Other mechanism | 814 | 3 (0.37) | 1 | 12 (1.47) | 2 |

**Panel B. Occurrence location**

| **Category** | **Category N** | **Alcohol positive, n (%)** | **Units, n** | **Psychotropic positive, n (%)** | **Units, n** |
| --- | --- | --- | --- | --- | --- |
| Residence | 29,529 | 176 (0.60) | 9 | 290 (0.98) | 11 |
| Road/street | 22,346 | 73 (0.33) | 12 | 33 (0.15) | 6 |
| Industrial/construction | 10,829 | 17 (0.16) | 5 | 18 (0.17) | 2 |
| Education | 1,144 | 0 (0.00) | 0 | 10 (0.87) | 2 |
| Commercial | 1,010 | 38 (3.76) | 9 | 0 (0.00) | 0 |
| Agricultural production | 852 | 2 (0.23) | 1 | 1 (0.12) | 1 |
| Recreation/sports | 1,372 | 11 (0.80) | 6 | 6 (0.44) | 4 |
| Other location | 1,229 | 13 (1.06) | 6 | 7 (0.57) | 2 |

*Positive status refers only to documented use by the injured person. Percentages use the corresponding outcome-category total as the denominator. Units, n is the number of reporting units contributing at least one positive record in that cell. These fields did not identify a uniform ascertainment source and are not equivalent to toxicologically confirmed exposure.*

# Supplementary Table S12. Annual mechanism and occurrence-location composition

**Panel A. Injury mechanism**

| **Category** | **2021 (N=9,467)** | **2022 (N=9,261)** | **2023 (N=11,432)** | **2024 (N=19,122)** | **2025 (N=19,029)** |
| --- | --- | --- | --- | --- | --- |
| Blunt injury | 6,463 (68.27) | 6,688 (72.22) | 9,157 (80.10) | 15,016 (78.53) | 15,144 (79.58) |
| Sharp injury | 937 (9.90) | 873 (9.43) | 853 (7.46) | 1,611 (8.42) | 1,418 (7.45) |
| Other mechanical injury | 1,083 (11.44) | 774 (8.36) | 647 (5.66) | 1,424 (7.45) | 1,381 (7.26) |
| Chemical/substance exposure | 298 (3.15) | 401 (4.33) | 269 (2.35) | 346 (1.81) | 254 (1.33) |
| Overexertion | 253 (2.67) | 196 (2.12) | 230 (2.01) | 394 (2.06) | 446 (2.34) |
| Thermal mechanism | 198 (2.09) | 151 (1.63) | 155 (1.36) | 207 (1.08) | 230 (1.21) |
| Other mechanism | 235 (2.48) | 178 (1.92) | 121 (1.06) | 124 (0.65) | 156 (0.82) |

**Panel B. Occurrence location**

| **Category** | **2021 (N=9,467)** | **2022 (N=9,261)** | **2023 (N=11,432)** | **2024 (N=19,122)** | **2025 (N=19,029)** |
| --- | --- | --- | --- | --- | --- |
| Residence | 4,112 (43.44) | 4,112 (44.40) | 4,897 (42.84) | 8,424 (44.05) | 7,984 (41.96) |
| Road/street | 2,608 (27.55) | 2,768 (29.89) | 4,079 (35.68) | 6,299 (32.94) | 6,592 (34.64) |
| Industrial/construction | 1,781 (18.81) | 1,525 (16.47) | 1,454 (12.72) | 3,011 (15.75) | 3,058 (16.07) |
| Education | 222 (2.34) | 126 (1.36) | 229 (2.00) | 291 (1.52) | 276 (1.45) |
| Commercial | 115 (1.21) | 147 (1.59) | 219 (1.92) | 304 (1.59) | 225 (1.18) |
| Agricultural production | 163 (1.72) | 122 (1.32) | 174 (1.52) | 218 (1.14) | 175 (0.92) |
| Recreation/sports | 195 (2.06) | 223 (2.41) | 229 (2.00) | 366 (1.91) | 359 (1.89) |
| Other location | 271 (2.86) | 238 (2.57) | 151 (1.32) | 209 (1.09) | 360 (1.89) |

*Values are n (%), with percentages calculated among all recorded injury visits in the corresponding year. The 2025 observation period ended on 25 November. These values describe annual case composition within the reporting network and are not population incidence rates.*
